# A Systems Biology-Based Approach to Uncovering the Molecular Mechanisms Underlying the Effects of Dragon's Blood Tablet in Colitis, Involving the Integration of Chemical Analysis, ADME Prediction, and Network Pharmacology

**DOI:** 10.1371/journal.pone.0101432

**Published:** 2014-07-28

**Authors:** Haiyu Xu, Yanqiong Zhang, Yun Lei, Xiumei Gao, Huaqiang Zhai, Na Lin, Shihuan Tang, Rixin Liang, Yan Ma, Defeng Li, Yi Zhang, Guangrong Zhu, Hongjun Yang, Luqi Huang

**Affiliations:** 1 Institute of Chinese Materia Medica, China Academy of Chinese Medical Sciences, Beijing, P.R. China; 2 National resource center for Chinese Materia Medica, China Academy of Chinese Medical Sciences, Beijing, P.R. China; 3 Beijing University of Chinese Medicine, Beijing, P.R. China; 4 Tianjin University of Traditional Chinese Medicine, Tianjin, P.R. China; 5 Yunnan Datang Hanfang Pharmacy co.ltd, Yunnan, P.R. China; Stavanger University Hospital, Norway

## Abstract

Traditional Chinese medicine (TCM) is one of the oldest East Asian medical systems. The present study adopted a systems biology-based approach to provide new insights relating to the active constituents and molecular mechanisms underlying the effects of dragon's blood (DB) tablets for the treatment of colitis. This study integrated chemical analysis, prediction of absorption, distribution, metabolism, and excretion (ADME), and network pharmacology. Firstly, a rapid, reliable, and accurate ultra-performance liquid chromatography-electrospray ionization-tandem mass spectrometry method was employed to identify 48 components of DB tablets. *In silico* prediction of the passive absorption of these compounds, based on Caco-2 cell permeability, and their P450 metabolism enabled the identification of 22 potentially absorbed components and 8 metabolites. Finally, networks were constructed to analyze interactions between these DB components/metabolites absorbed and their putative targets, and between the putative DB targets and known therapeutic targets for colitis. This study provided a great opportunity to deepen the understanding of the complex pharmacological mechanisms underlying the effects of DB in colitis treatment.

## Introduction

Traditional Chinese medicine (TCM) is one of the oldest medical systems of health care, and it has been used in East Asian countries such as China, Japan, and Korea for thousands of years [1], [2]. However, acceptance of traditional Chinese medicines within Western biomedical practice has been restricted by a lack of knowledge of the active compounds involved, and their therapeutic mechanisms of action [3]. TCM is characterized by the usage of multi-component, multi-target agents that collectively modulate molecular networks. Recently, network pharmacology has provided a means to improve understanding of the molecular mechanisms underlying the therapeutic effects of traditional Chinese medicines [3]–[5]. TCM chemical databases such as TCM Database@Taiwan [6] and HerbBioMap database [7] and some literature generally provide the main sources of information on the chemical profiles of traditional Chinese medicines. However, these are not always accurate because the chemical profiles of traditional Chinese medicines can vary significantly, depending on the geographical origin of the materials used, the harvest time, pretreatments, and manufacturing processes employed. In addition, most TCM formulations are taken orally, estimation of intestinal absorption and cytochrome P450 metabolism provide more in-depth insights into their therapeutic mechanisms [8], [9]. However, experimental determination using these systems can be costly and time-consuming. To obtain a rapid estimation of human absorption and metabolism, high throughput screening methods using Caco-2 cell monolayers and P450 enzymes provide the most advanced *in vitro* approaches to assessing new chemical entities [10]–[12]. Thus, the development of rapid high-throughput approaches to studying TCM compositions and mechanisms, combining chemical analysis, prediction of absorption, distribution, metabolism, and excretion (ADME), and network pharmacology is required.

According to the Pharmacopoeia of the People's Republic of China, commercially available longxuejie or dragon's blood (DB) includes plant resins from four species; *Dracaena* spp., *Daemonorops* spp., *Croton* spp., and *Pterocarpus* spp., and has long been used as an ethnomedicine in China to invigorate blood circulation in the treatment of traumatic injuries, blood stasis, and pain [13], [14]. DB has also been used to treat chronic colitis in recent decades and many clinical studies have reported good therapeutic effects [15]–[17]. Chemically, flavonoids, phenols, steroides, and terpenoids have been identified as the main constituents of DB [18]–[22]. Some analytical methods have been developed to identify and characterize DB composition and assess its quality [23]–[25]. Pharmacologic studies of these components have identified a wide variety of actions, including anti-inflammatory, antiulcer, antimicrobial, hemostatic, and analgesic activities, which could contribute to their efficacy in the treatment of chronic colitis [26]–[28]. However, the precise active constituents of DB that have benefits in chronic colitis, and their molecular mechanisms of action, are still unclear. In particular, the lack of elucidation of the ingredient-target network has hindered understanding of the molecular mechanisms of DB in chronic colitis treatment.

**Figure 1:**
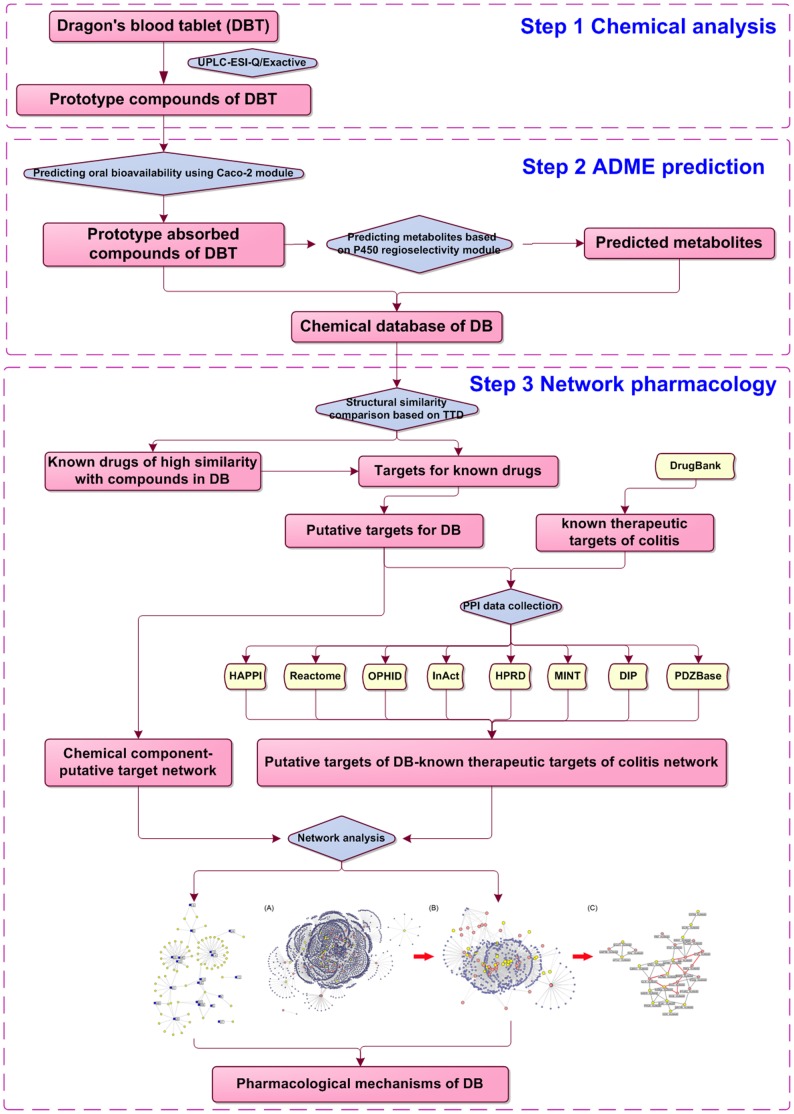
A schematic diagram of this systems biology-based investigation into the molecular mechanisms of DB tablet for colitis by integrating chemical analysis, ADME prediction and network pharmacology.

The present study employed high-throughput analysis, *in silico* ADME models, and a network pharmacology technique to investigate the active constituents of DB and their actions on molecular networks in chronic colitis, as shown in **Figure 1**.

## Materials and Methods

### 1. Reagents and chemicals

High performance liquid chromatography (HPLC)-grade acetonitrile, formic acid, and methanol were obtained from Merck (Darmstadt, Germany). Water was purified using a Milli-Q system (Millipore, Billerica, MA, USA). 7,4′-Dihydroxyflavan, loureirin A, loureirin B, dracaenin A and pterostilbene standards were purchased from the National Institute for the Control of Pharmaceutical and Biological Products (Beijing, China). The purities of all standards were no less than 98% and suitable for liquid chromatography-tandem mass spectrometry (LC-MS/MS) analysis. DB enteric-coated tablets were supplied from Yunnan Datang Hanfang Pharmaceutical Co. Ltd. (Yunnan, China).

### 2. Preparation of samples and standard solution

Six different batches of DB enteric-coated tablets were pulverized with a 60 mesh, respectively. Each sample of this powder (0.1 g) was weighed precisely and ultrasonically extracted in 10 ml methanol for 20 min at room temperature. The solution was centrifuged at 12000 rpm for 10 min, and then filtered through 0.22 µm nylon membrane filters. The filtrate was analyzed directly by UPLC-ESI-MS/MS, as described in section 2.3. At the same time, a stock solution containing five standards (7,4′-dihydroxyflavan, loureirin A, loureirin B, pterostilbene, and dracaenin A) was prepared in methanol. All solutions were stored at 4°C prior to analysis.

### 3. Instrument and UPLC-ESI-MS/MS conditions

UPLC was performed on a Dionex UltiMate 3000 system (Dionex Corporation, Sunnyvale, USA) equipped with a quaternary pump, an online vacuum degasser, an autosampler, and an automatic thermostatic column oven. A Thermo Hyper Gold C18 column was used (100×2.1 mm, 1.9 µm) at 30°C with a flow rate of 0.4 ml/min and an injection volume of 5 µl. The mobile phase was a mixture of 0.1% formic acid in water (A) and acetonitrile (B). The mobile phase gradient program was 5% B, 0–3 min; 5–95% B, 3–20 min; 95% B, 20–25 min. High-resolution accurate-mass full scan LC-MS and LC-MS/MS analyses were performed using Thermo Q-Exactive (Thermo Fisher Scientific, Bremen, Germany). Full scans were acquired in the mass analyzer at 100–1500 m/z with a resolution of 70000 in both positive and negative ion modes, and MS/MS scans were obtained with a resolution of 17500 using a normalized collision energy of 30% for high-energy collisional dissociation fragmentation. Based on the best response for most compounds, the final parameters were set as follows: spray voltage, 3.2 kV in the positive mode and 3.0 kV in the negative mode; capillary temperature, 350°C; sheath gas pressure, 35 arbitrary units; auxiliary gas pressure, 10 arbitrary units; and heater temperature, 300°C. Xcalibur 2.2 software (Thermo Fisher Scientific) was used for data acquisition.

### 4. Prediction of absorbed constituents and their metabolites using in silico ADME models

Structural information (*.mol or *.sdf files) relating to the DB components(identified by UPLC-ESI-MS/MS) were downloaded from ChemSpider (http://www.chemspider.com/). ADME evaluation of these constituents was carried out using ACD/Percepta software 5.07 (ACD/Labs, Toronto, Canada), including the passive intestinal permeability of Caco-2 module and the P450 regioselectivity module to predict their oral bioavailability. The apparent permeability coefficient (Papp) of one constituent was greater than 9.0×10^6^ cm/s, which indicated good absorption characteristics. The score and the reliability of metabolic reaction were set at greater than 0.6 and 0.5, respectively, in order to increase prediction credibility.

### 5. Known therapeutic targets for colitis treatment

Known therapeutic targets were obtained from the DrugBank database [29] (http://www.drugbank.ca/, version: 3.0). We only included drug-target interactions where the drugs were approved by the Food and Drug Administration, USA (FDA) for the treatment of colitis, with human gene/protein targets. To facilitate data analysis, all protein identification codes were converted to a common UniProtKB-Swiss-Prot code, and detailed target information is provided in the supplementary **Table S1.**

### 6. PPI data collection

PPI data were imported from eight existing PPI databases, including the Human Annotated and Predicted Protein Interaction Database (HAPPI) [30], Reactome [31], Online Predicted Human Interaction Database (OPHID) [32], InAct [33], Human Protein Reference Database (HPRD) [34], Molecular Interaction Database (MINT) [35], Database of Interacting Proteins (DIP) [36], and PDZBase [37]. Detailed information on these PPI databases is provided in the supplementary **Table S2**.

### 7. Pharmacological mechanism analysis

#### 7.1. Prediction of putative DB targets

As described in our previous study [38], we used the Drug Similarity Search tool in the Therapeutic Targets Database [39] (TTD, http://xin.cz3.nus.edu.sg/group/cjttd/ttd.asp, Version 4.3.02, released on Aug 25th 2011) to screen drugs similar to DB via structural similarity comparisons. We only selected drugs with a high structural similarity score (>0.85, similar to very similar) with the potentially absorbed DB constituents and their metabolites. The therapeutic targets of these similar drugs were also included as putative DB targets.

#### 7.2. Network construction and analysis

The absorbed DB components and their metabolites, their putative targets, and known therapeutic targets for colitis treatment were used to construct a chemical component-putative target network, and a putative DB targets-known colitis therapeutic targets PPI network, respectively. PPI data were obtained from eight existing PPI databases, as described in section 2.6. Navigator software (Version 2.2.1) and Cytoscape (Version 2.8.1) were utilized to visualize the networks.

For each node “i” in these two networks, we defined four measures of topology: (1) “degree,” defined as the number of links to node i; (2) “betweenness,” defined as the capacity of node i to be located in the shortest communication paths between different pairs of nodes in the network [40]. The betweenness centrality of node i is computed as following formula:
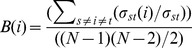
where 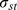 denotes the number of the shortest paths between node s and node t in the PPI network, 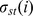 denotes the number of the shortest paths across node i between node s and node t, and N is the total number of nodes in the PPI network. This property correlates more closely with essentiality than connectivity, exposing critical nodes that usually belong to the group of scaffold proteins or proteins involved in crosstalk between signal pathways (called bottlenecks) [41]. (3) “closeness,” defined as the inverse of the sum of node i distances to all other nodes. Closeness can be regarded as a measure of how long it would take to spread information from node i to all other nodes sequentially. Degree, betweenness and closeness centralities correlate with a protein's topological importance in the PPI network [40]. (4) K-core analysis is an iterative process in which nodes were removed from the network in order of the least-connected [42]. The core of maximum order is defined as the main (highest) k-core in the network. A k-core sub-network of the original network can be generated by recursively deleting vertices from the network whose degree is less than k. This results in a series of sub-networks that gradually reveal the globally central region of the original network. On this basis, “k value” is used to measure the centrality of node i.

#### 7.3 Pathway enrichment analysis for the major putative DB targets and the major known therapeutic targets for colitis

We performed pathway enrichment analysis using pathway data obtained from the FTP service of KEGG [43] (Kyoto Encyclopedia of Genes and Genomes, http://www.genome.jp/kegg/, Last updated: Oct 16, 2012).

## Results and Discussion

### 1. Optimization of UPLC-ESI-MS/MS conditions

**Figure 2:**
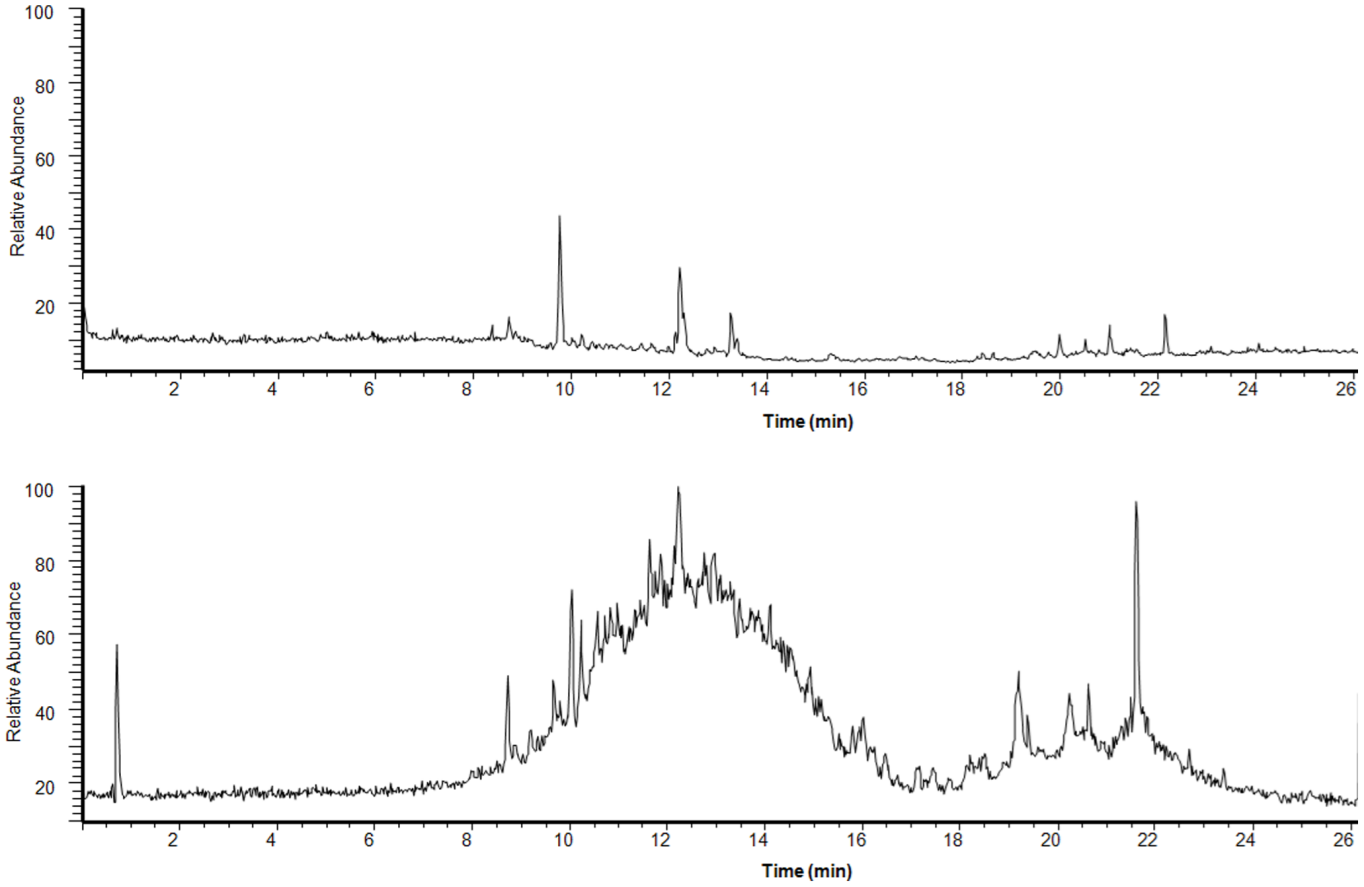
UPLC-ESI-MS/MS of DB in positive ion mode (A) and negative ion mode (B).

To obtain reliable chromatographic results and appropriate ionization, four mobile phase systems of acetonitrile-water, methanol-water, acetonitrile-acid aqueous solution, and methanol-acid aqueous solution were tested and compared. The results suggested that acetonitrile-formic acid aqueous solution was superior to the others. Different concentration of formic acid aqueous solution (0.05%, 0.1%, and 0.5%) were investigated, indicating that use of 0.1% formic acid aqueous solution produced the optimal peak shape and reduced peak tailing, as well as increasing ion response for most compounds. Meanwhile, the process of gradient elution was optimized to obtain better separation over shorter time periods. The MS parameters were optimized to improve ion intensity. These optimized parameters were described in section 2.3. Base peak chromatograms of DB in positive and negative ion modes are shown in **Figure 2**.

### 2. Screening and identification of DB using UPLC-ESI-MS/MS

**Figure 3:**
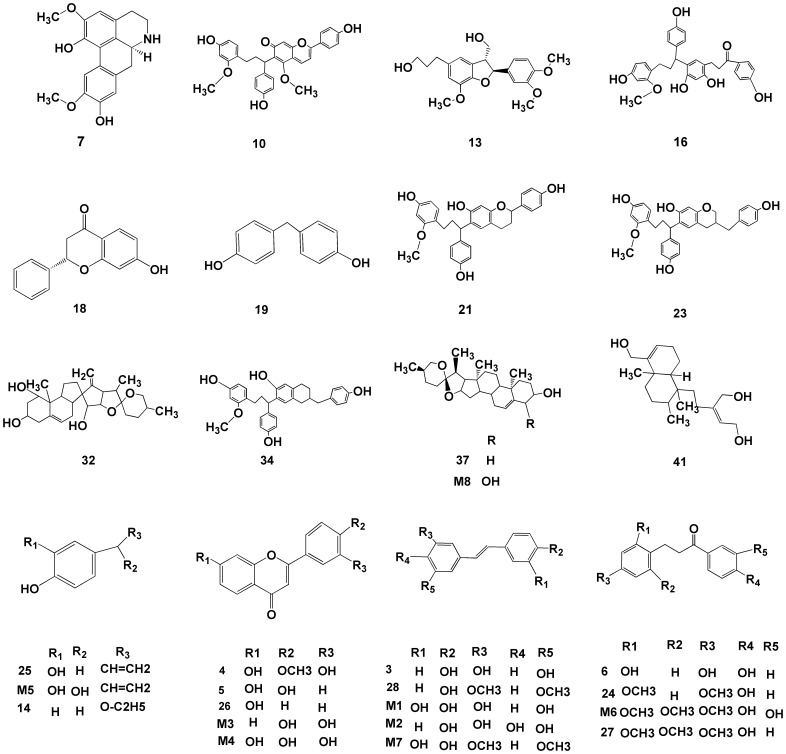
The structures of the 20 potentially absorbed DB constituents and 8 metabolites. **3.** 3,5,4′-trihydroxystilbene; **4.** 4′-methoxy-3′7-dihydroxyflavan; **5.** 7,4′-dihydroxyflavan; **6.** 2,4,4′-trihydroxydihydrochalcone; **7.** norisoboldine; **10.** dracaenin A; **13.** 3,4-*o*-dimethylcedrusin; **14.** ethyl 4-hydroxybenzoate; **16.** cochinchinenin; **18.** (2*S*)-7-hydroxyflavanone; **19.** 4,4′-dihydroxydiphenylmethane; **21.** homoisosocotrin-4-ol; **24.** loureirin A; **25.** 3,4-dihydroxy-allylbenzene; **26.** 7-hydroxyflavanone; **27.** loureirin B; **28.** pterostilbene; **32.** dracaenol B; **34.** cochinchinenin E; **37.** diosgenin; **41.** bincatriol. M1 and M2 were metabolites of compound **3**; M3, M4, M5, M6, M7, and M8 were metabolites of compounds **4**, **5**, **25**, **27**, **28**, and **37**, respectively.

**Figure 4:**
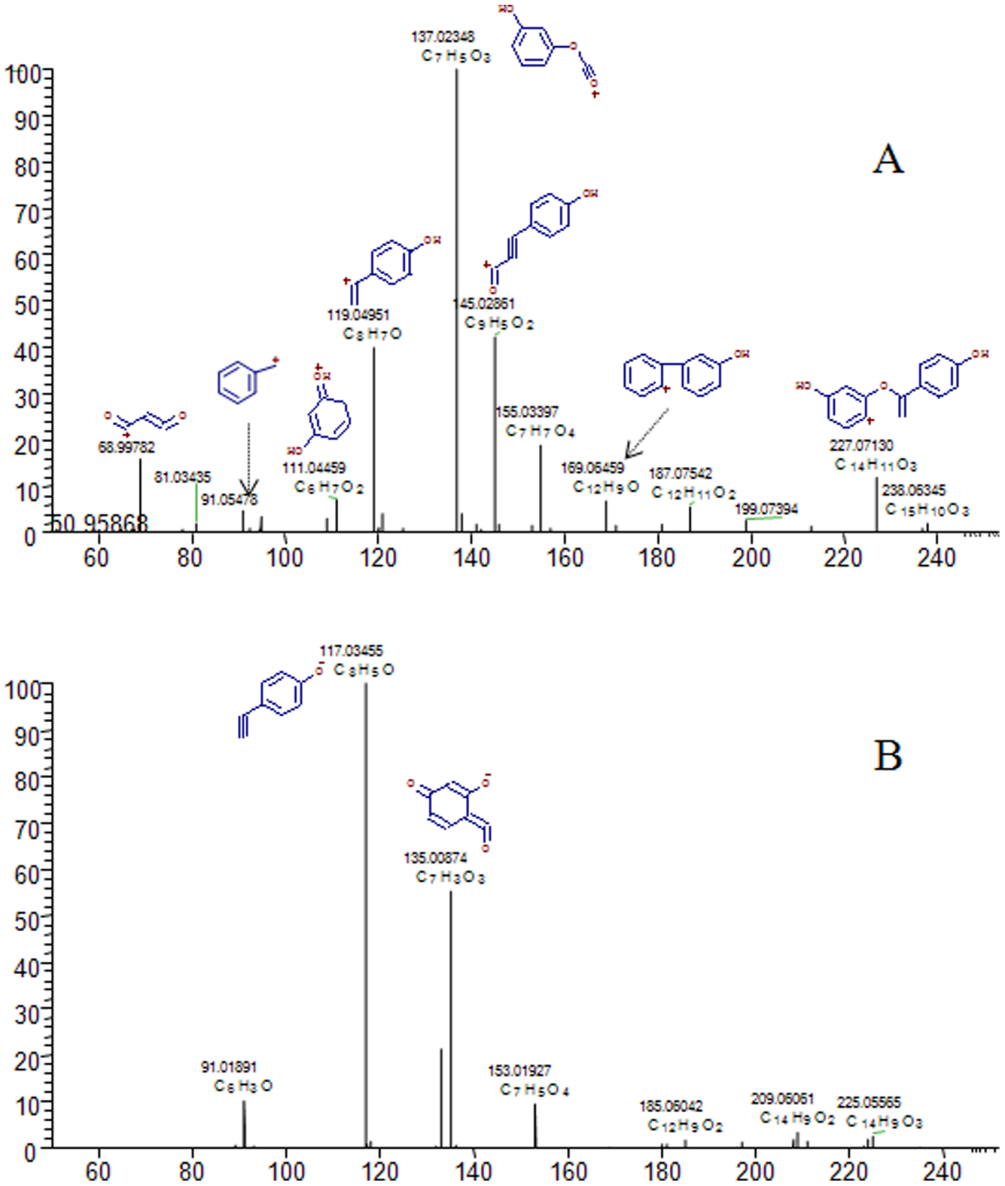
The MS/MS spectrum and possible fragmentation pathways of 7,4′-dihydroxyflavan.

Recently, the UPLC-Q-Exactive system was reported to provide a rapid, reliable and accurate technique for identification of herbal constitutions, because of its separation efficiency, high resolution and high mass accuracy [44]–[46]. In the present study, a total of 48 components of DB were identified. These were mainly flavonoids, such as flavones, flavonols, flavanones, chalcones, and isoflavones, as listed in **Table 1**. For some compounds (7,4′-dihydroxyflavan, loureirin A, loureirin B, pterostilbene, and dracaenin A), purified standards were available and these were identified by comparing sample retention time and accurate mass with that of the standard. For compounds where standards were unavailable, identification was based on accurate mass and tandem mass spectra. The molecular formula was established by high-accuracy quasi-molecular ion such as [M-H]^−^, [M+CH_3_COO]^−^, [M+H]^+^, and [M+Na]^+^ within a mass error of 5.0 ppm and fractional isotope abundance. The most rational molecular formula was identified using chemical or herbal databases, such as Chemspider (www.chemspider.com) and TCM Database@Taiwan (http://tcm.cmu.edu.tw). When several isomers were matched, a compound that had previously been identified in DB was considered more likely to be correct. Finally, ion fragments were used to provide further confirmation of the chemical structure. The structures of the main constitutions of DB are shown in **Figure 3**. For example, 7,4′-dihydroxyflavan (compound **5**) was firstly identified by comparing its retention time and accurate mass with that of the appropriate standard. Then, the MS/MS spectrum and possible fragmentation pathways of 7,4′-dihydroxyflavan were depicted in **Figure 4**. Only [M+H]^+^ and [M–H]^−^ were observed in positive and negative ion modes, respectively. The loss of 128 Da and 136 Da were the characteristic neutral loss of 7,4′-dihydroxyflavan in positive and negative ion modes.

**Table 1:** MS data of ESI-MS spectra and identification of the Longxuejie enteric-coated tablet.

No.	RT (Measured)	m/z (Measured)	m/z (Expected)	m/z (Delta, ppm)	polarity	Formula	Compound Name	Papp (10^−6^ cm/s)
1	8.11	311.11395	311.11363	1.046	negative	C_15_H_20_O_7_	3,4-dihydroxyallylbenzene 4-O—D-Glucopyranoside	3.8494
2	8.38	285.07592	285.07575	0.594	positive	C_16_H_12_O_5_	unknown	—
		283.06137	283.0612	0.606	negative			
3	8.68	229.08608	229.08592	0.702	positive	C_14_H_12_O_3_	3,5,4'-trihydroxystilbene	148.9186
		227.07154	227.07137	0.752	negative			
4	8.68	273.07713	273.07685	1.054	negative	C_15_H_14_O_5_	4'-methoxy-3'7-dihydroxyflavan	115.6472
5	8.72	255.06525	255.06519	0.241	positive	C_15_H_10_O_4_	7,4'-dihydroxyflavan	137.4393
		253.05074	253.05063	0.408	negative			
6	8.74	259.09663	259.09649	0.563	positive	C_15_H_14_O_4_	2,4,4'-Trihydroxydihydrochalcone	94.5322
		257.08211	257.08193	0.707	negative			
7	8.83	314.13891	314.13868	0.703	positive	C_18_H_19_NO_4_	norisoboldine	30.448
		312.12448	312.12413	1.11	negative			
8	9.27	149.06084	149.0608	0.219	negative	C_9_H_10_O_2_	unknown	—
9	9.66	269.08209	269.08193	0.594	negative	C_16_H_14_O_4_	unknown	—
10	9.78	525.19107	525.19078	0.558	positive	C_32_H_28_O_7_	dracaenin A	134.7937
		523.1766	523.17623	0.709	negative			
11	10.02	273.11223	273.11214	0.359	positive	C_16_H_16_O_4_	unknown	—
		271.09769	271.09758	0.385	negative			
12	10.21	303.1228	303.1227	0.339	positive	C_17_H_18_O_5_	unknown	—
		301.10831	301.10815	0.531	negative			
13	10.21	392.20704	392.20676	0.696	positive	C_21_H_26_O_6_	3,4-O-dimethylcedrusin	149.4172
14	10.21	167.0704	167.07027	0.788	positive	C_9_H_10_O_3_	Ethyl 4-hydroxybenzoate	216.6625
15	10.57	287.09152	287.0914	0.414	positive	C_16_H_14_O_5_	unknown	—
		285.07703	285.07685	0.638	negative			
16	10.73	532.23343	532.23298	0.849	positive	C_31_H_30_O_7_	cochinchinenin	21.059
		513.19218	513.19188	0.587	negative			
17	10.95	301.10837	301.10815	0.733	negative	C_17_H_18_O_5_	unknown	—
18	11.2	241.08608	241.08592	0.665	positive	C_15_H_12_O_3_	(2S)-7-hydroxyflavanone	227.3156
		239.07151	239.07137	0.616	negative			
19	11.45	199.07646	199.07645	0.057	negative	C_13_H_12_O_2_	4,4'-Dihydroxydiphenylmethane	201.7499
20	11.64	529.22251	529.22208	0.817	positive	C_32_H_32_O_7_	unknown	—
		527.20758	527.20753	0.102	negative			
21	11.65	511.21352	511.21261	1.776	negative	C_32_H_32_O_6_	homoisosocotrin-4-ol	17.1673
22	11.86	283.09773	283.09758	0.507	negative	C_17_H_16_O_4_	unknown	—
23	12	516.23823	516.23806	0.321	positive	C_31_H_30_O_6_	socotrin 4-ol	7.5670
24	12.13	287.12781	287.12779	0.077	positive	C_17_H_18_O_4_	Loureirin A	228.3831
		285.11337	285.11323	0.492	negative			
25	12.13	151.07542	151.07536	0.442	positive	C_9_H_10_O_2_	3,4-Dihydroxy-allylbenzene	140.4007
26	12.23	227.10674	227.10666	0.351	positive	C_15_H_14_O_2_	7-hydroxyflavanone	227.3156
27	12.23	317.13839	317.13835	0.136	positive	C_18_H_20_O_5_	Loureirin B	227.8266
		315.12406	315.1238	0.833	negative			
28	12.93	257.11728	257.11722	0.227	positive	C_16_H_16_O_3_	pterostilbene	236.2931
		255.10277	255.10267	0.396	negative			
29	13	885.45033	885.44894	1.572	negative	C_44_H_70_O_18_	26-O—D-Glucopynanosyl-furostan-5,25(27)-diene-1,3,22,26-tetrol-1-O-[-L-rhamnopyranosyl(12)]—L-Arabinopyranoside	0.0073
30	13.25	167.0704	167.07027	0.799	positive	C_9_H_10_O_3_	unknown	—
31	14.08	265.14798			negative		unknown	—
32	14.39	462.32139	462.3214	−0.029	positive	C_27_H_40_O_5_	dracaenol B	141.9713
33	14.90	571.23383			negative		unknown	—
34	14.91	527.24323	527.24282	0.786	positive	C_33_H_34_O_6_	cochinchinenin E	8.2809
35	15.77	532.92554			negative		unknown	—
36	15.92	325.18451	325.18569	−3.642	negative	C_14_H_29_O_8_	unknown	—
37	16.27	415.32104	415.32067	0.894	positive	C_27_H_42_O_3_	Diosgenin	112.3202
38	16.44	325.18448	325.18569	−3.734	negative	C_14_H_29_O_8_	unknown	—
39	17.44	339.20010	339.20134	−3.668	negative	C_15_H_31_O_8_	unknown	—
40	19.18	698.91071			negative		unknown	—
41	19.95	323.25817	323.25807	0.292	positive	C_20_H_34_O_3_	bincatriol	172.7858
42	20.25	445.29987			negative		unknown	—
43	20.49	628.19489			positive		unknown	—
44	20.63	383.18988	383.19117	−4.807	negative	C_16_H_31_O_10_	unknown	—
45	21	461.32938			positive		unknown	—
46	21.6	116.92857			negative		unknown	—
47	22.14	394.3465	394.34415	4.577	Positive	C_25_ H_46_ O_3_	unknown	—
48	24.99	427.3939	427.39344	1.082	positive	C_30_H_50_O	lupeol	0.0386

Papp: apparent permeability coefficient at the indicated concentrations in the basolateral-to-apical (B-A) direction across a Caco-2 cell monolayer.

### 3. Prediction of absorbed DB constituents and their metabolites by in silico ADME models

Quantitative structure-permeability relationships (QSPerR) have been established for permeability across Caco-2 monolayers (Papp) by the application of new molecular descriptors [47]. Among them, the passive Caco-2 absorption model provides a useful drug discovery tool by predicting human oral absorption of new chemical entities [48]–[49]. Yazdanian *et al*. [50] reported that compounds with Papp values less than 0.4×10^−6^ cm/s exhibited very poor oral absorption, whereas compounds with Papp values greater than 7×10^–6^ cm/s had excellent oral absorption. The present study employed an *in silico* passive Caco-2 absorption model within the ACD/Percepta software to identify DB constituents with excellent oral absorption values (greater than 7×10^−6^ cm/s) and the results were shown in **Table 1**. Subsequently, the ACD/Percepta software P450 regioselectivity module was applied to predict the metabolites of these well-absorbed DB constituents. These results were listed in **Table 2** and the structures of the metabolites were shown in **Figure 3**.

**Table 2:** Prediction of the score, reliability, reaction site and reaction type of excellent oral absorbed constituents of LXJ by the P450 regioselectivity module in ACD/Percepta, respectively.

No.	Compound No	Compound name	Score	Reliability	Reaction site	Reaction type	Metabolizing enzymes
M1	3	3,5,4'-trihydroxyStilbene	0.94	0.86	3	Aromatic Hydroxylation	CYP1A2
			0.94	0.86	7	Aromatic Hydroxylation	CYP1A2
M2	3	3,5,4'-trihydroxyStilbene	0.65	0.87	14	Aromatic Hydroxylation	CYP1A2
M3	4	4'-methoxy-3'7-dihydroxyflavan	0.92	0.78	21	O-dealkylation	CYP1A2
M4	5	7,4'-Dihydroxyflavan	0.92	0.79	13	Aromatic Hydroxylation	CYP1A2
			0.92	0.79	15	Aromatic Hydroxylation	CYP1A2
M5	25	3,4-Dihydroxy-allylbenzene	0.85	0.74	4	Aliphatic Hydroxylation	CYP2C19
			0.91	0.86	4	Aliphatic Hydroxylation	CYP2C9
M6	27	Loureirin B	0.74	0.5	18	Aromatic Hydroxylation	CYP1A2
			0.74	0.5	19	Aromatic Hydroxylation	CYP1A2
M7	28	pterostilbene	0.90	0.74	10	Aromatic Hydroxylation	CYP1A2
			0.90	0.74	13	Aromatic Hydroxylation	CYP1A2
M8	37	Diosgenin	0.64	0.52	24	Aliphatic Hydroxylation	CYP3A4

### 4. Analysis of DB pharmacological mechanisms in colitis

In order to analyze the synergistic effects and pharmacological mechanisms of DB in colitis, we constructed a chemical component-putative target network, and a putative DB targets-known colitis therapeutic targets PPI network. Detailed information relating to these similar drugs and putative targets is provided in supplementary **Table S3**. The performance of this prediction method was evaluated in our previous study [34].

#### 4.1. DB chemical component-putative target network

**Figure 5:**
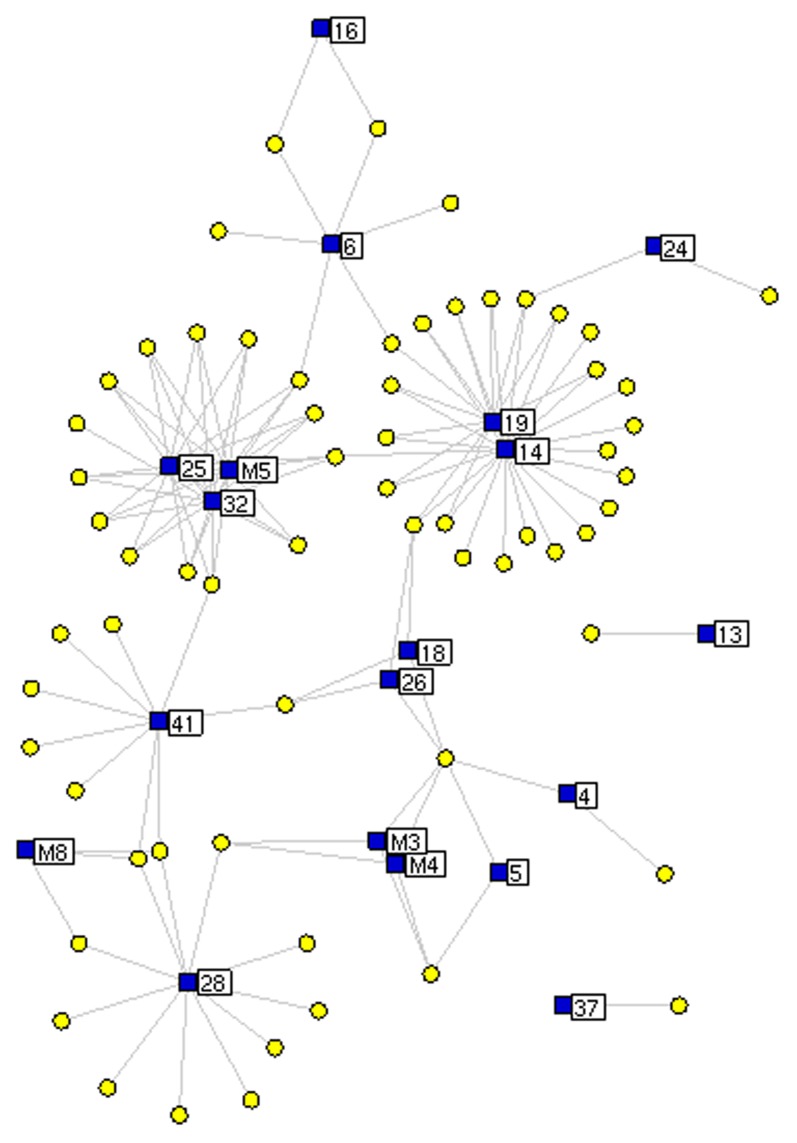
DB chemical component-putative target network. Blue squares refer to the DB compounds and metabolites; yellow spherical nodes refer to the predicted targets.

As shown in **Figure 5**, this network consisted of 67 nodes (15 absorbed components, 4 metabolites and 48 putative targets) and 120 edges. The mean number of putative targets per chemical component/metabolite was 2.53. The absorbed DB component 14 and the metabolite M5 of compound **25** (3,4-dihydroxy-allylbenzene) both had the highest degree distributions, and hit 23 and 14 putative targets, respectively. Since chemical components or metabolites with a higher degree in the network have been demonstrated to be more pharmacologically important, and our data indicated that components 25, 32, and the M5 metabolite had the shortest paths between each other, they may play major roles in this pharmacological network, with synergistic effects.

#### 4.2. Putative DB targets-known colitis therapeutic targets PPI network

**Figure 6:**
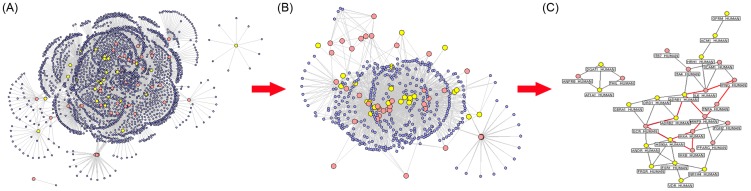
Putative DB targets-known colitis therapeutic targets protein-protein interaction (PPI) network. (A) The network between all targets and other human proteins. (B) The network of hub proteins in network (A). (C) The network of the major putative DB targets and the major known colitis therapeutic targets in network (B). Yellow spherical nodes indicate the putative targets; pink spherical nodes indicate the known therapeutic targets; purple spherical nodes indicate other human proteins that interact with putative targets or known therapeutic targets. Red edges in (C) indicate the PPIs of targets involved in the NOD-like receptor signaling pathway.

Putative DB targets-known colitis therapeutic targets PPI network was constructed using PPIs between putative DB targets and known colitis therapeutic targets. As shown in **Figure 6A**, this network consisted of 2397 nodes and 4005 edges. According to the previous study of Li et al. [51], if the degree of a node is more than 2 fold of the median degree of all nodes in a network, such node is believed to play a critical role in the network structure, and can be treated as a hub node. Thus, we identified 463 hub proteins. Then, the PPI network of these hub proteins was constructed using the direct PPIs between them. As a result, this network consisted of 463 nodes and 1574 edges (**Figure 6B**). In addition, we calculated four topological feature values of all 463 nodes in the PPI network of hub proteins according to the definition described in section 2.7.2. We defined a hub protein, the four topological feature values of which were all larger than the corresponding median values, as a major hub protein. After the calculation, the median values of the four topological features were 3, 0.06, 31.30, and 3 for “degree,” “betweenness,” “closeness,” and “k value,” respectively. Therefore, we determined that hub proteins with “degree” >3, “betweenness” >0.06, “closeness” >31.30, and “k value” >3 were major hubs. As a result, 106 proteins were identified as major hubs, including 18 putative DB targets and 16 known therapeutic targets for colitis. Detailed information on the topological features of these major hubs is provided in supplementary **Table S4**.

To investigate the pharmacological mechanisms of DB in colitis further, we analyzed the direct PPIs between the major putative DB targets and the major known therapeutic targets for colitis. As shown in **Figure 6C**, this network contained 34 nodes and 42 edges. According to the results of enrichment analysis based on the KEGG pathway [43], these major target proteins were frequently involved in the NOD-like receptor signaling pathway (KEGG ID: map04621). The intracellular NOD-like receptor (NLR) family contains more than 20 members in mammals and plays a pivotal role in the recognition of intracellular ligands. NOD1 and NOD2, two prototypic NLRs, sense the cytosolic presence of bacterial peptidoglycan fragments that have escaped from endosomal compartments, driving the activation of NF-kappa-B and MAPK, cytokine production, and apoptosis [52]. Accumulating studies have demonstrated that several members of the NOD-like receptor signaling pathway, such as heat shock protein 90 (Hsp90) [53], interleukin-6 (IL-6) [54], tumor necrosis factor alpha (TNF-α) [55], NF-kappa-B inhibitor kinase alpha (IKKA) [56], IKKB [56], and adrenergic receptors [57], may be associated with the pathogenesis of colitis. Of these proteins, Hsp90, ADRB1, and ADRB2 were identified as putative DB targets. Moreover, a number of putative DB targets also interacted with members of the NOD-like receptor signaling pathway, as shown in **Fig. 6C**. These findings suggested that the therapeutic effects of DB on colitis may involve this pathway.

## Conclusion

High-throughput analysis, *in silico* ADME prediction, and network pharmacology techniques have recently emerged as powerful tools to provide new insights into the active compounds and the molecular mechanisms involved in the therapeutic actions of traditional Chinese medicines. In this study, a rapid, sensitive, and high-throughput analytical method using UPLC-ESI-MS/MS was firstly developed to characterize 48 constituents and accurately identify 24 constituents of DB tablet. Then, 22 components of DB tablet were predicted to be absorbed by an *in silico* passive absorption model, based on the Caco-2 cell monolayer. Eight metabolites were predicted using a computational module of P450 regioselectivity. Finally, these compounds were predicted to interact with 26 putative targets. Using this information, pharmacological networks were built to visualize the synergistic interactions between DB components and their targets. Furthermore, a PPI network of putative DB targets and known therapeutic targets for colitis was constructed to pinpoint the key targets and pathways. This approach offered a valuable opportunity to deepen understanding of the pharmacological mechanisms of DB in colitis.

In summary, this research provided a more accurate analysis of DB constituents, compared to using TCM databases. Consideration of ADME using the passive absorption and P450 metabolism modules *in silico* predicted the *in vivo* situation more closely. Finally, network pharmacology analysis was applied effectively and helped to interpret the essence of “synergy” in Chinese medicine. This provided a new way to identify the key candidate targets and possible molecular pathways utilized by DB in the treatment of colitis.

## Supporting Information

Table S1 The detailed target information for colitis.
(XLSX)

Table S2 The detailed PPI information of colitis based on eight existing PPI databases.
(XLSX)

Table S3 Detailed information relating to these similar drugs and putative targets for DB curing colitis.
(XLSX)

Table S4 Detailed information on the topological features of these major hubs including 18 putative DB targets and 16 known therapeutic targets for colitis.
(XLSX)
